# Insight into microRNA regulation by analyzing the characteristics of their targets in humans

**DOI:** 10.1186/1471-2164-10-594

**Published:** 2009-12-10

**Authors:** Zihua Hu

**Affiliations:** 1 Center for Computational Research, New York State Center of Excellence in Bioinformatics & Life Sciences, Department of Biostatistics, Department of Medicine, State University of New York (SUNY), Buffalo, NY 14260, USA

## Abstract

**Background:**

microRNAs (miRNAs) are believed to regulate their targets through posttranscriptional gene regulation and have the potential to silence gene expression via multiple mechanisms. Despite previous advances on miRNA regulation of gene expression, little has been investigated from a genome scale.

**Results:**

To gain new insight into miRNA regulation in humans, we used large scale data and carried out a series of studies to compare various features of miRNA target genes to that of non-miRNA target genes. We observed significant differences between miRNA and non-miRNA target genes for a number of characteristics, including higher and broader mRNA expression, faster mRNA decay rate, longer protein half-life, and longer gene structures. Based on these features and by analyzing their relationships we found that miRNA target genes, other than having miRNA repression, were most likely under more complex regulation than non-miRNA target genes, which was evidenced by their higher and broader gene expression but longer gene structures. Our results of higher and broader gene expression but fast mRNA decay rates also provide evidence that miRNA dampening of the output of preexisting transcripts facilitates a more rapid and robust transition to new expression programs. This could be achieved by enhancing mRNA degradation through an additive effect from multiple miRNA targeting.

**Conclusion:**

Genome-scale analysis on the nature of miRNA target genes has revealed a general mechanism for miRNA regulation of human gene expression. The results of this study also indicate that miRNA target genes, other than having miRNA repression, are under more complex gene regulation than non-miRNA target genes. These findings provide novel insight into miRNA regulation of human gene expression.

## Background

miRNAs, which were first discovered in *Caenorhabditis elegans *as post-transcriptional regulators of genes involved in developmental timing [1,2], are small non-coding RNAs of ~23 nucleotides. They are now recognized as one of the major regulatory gene families, playing important roles in almost every cellular process in animals, plants and viruses [3-5]. In animals, this includes regulation of developmental timing and signaling pathways, apoptosis, metabolism, myogenesis and cardiogenesis, brain development [3], and human pathologies [6-8].

Although the mechanism by which miRNAs regulate gene expression remains under debate, miRNAs mainly mediate gene regulation post-transcriptionally via translational repression and reduction of mRNA stability by forming miRNA-mRNA pairs to their target genes. In vertebrates, most miRNAs pair imperfectly with the 3' untranslated regions (3'UTRs) of their targets, with a contiguous and perfect base pairing of the miRNA nucleotides 2-7 at the 'seed' region, providing pairing specificity [9-11]. Whereas miRNAs repress translation of target mRNAs by inhibiting translation initiation [12-14], blocking translation elongation [15,16], or promoting premature dissociation of ribosomes [16], they induce significant degradation of mRNA targets by mRNA deadenylation, decapping, and 5' → 3' exonucleolytic degradation. The latter have been widely demonstrated from animal studies [17,18], cultured cells [19-21], and microarray analysis [19,20,22,23]. Contradicting these mechanisms, it has been recently discovered that miRNA have the potential to activate translation under certain conditions [24-26] and the ability to switch from translational repression to translational activation in cell-cycle-arrested cells [27-29].

It has been found that individual miRNA can mildly down-regulate hundreds of targets by direct or indirect effects, providing a mechanism of fine-tuning for gene expression. The first demonstration of this came from a microarray study in which introduction of an exogenous miRNA into human HeLa cells downregulated a large number of target mRNAs [19]. Consistently, depletion of the miRNA machinery proteins destabilized around 20% of transcripts expressed in *Drosophila *[30]. In Zebrafish, miR-430 was found to facilitate the deadenylation and clearance of several hundred target mRNAs, most of which were maternally expressed and accumulated in the absence of miR-430 [18]. In another study that employed a proteomic approach to measure changes in the synthesis of proteins in response to miRNA transfection or endogenous miRNA knockdown, a single miRNA could repress the production of hundreds of proteins, but the repression was relatively mild [31]. It has also been demonstrated that some miRNAs dampen the output of preexisting but unwanted transcripts to facilitate a more rapid and robust transition to new expression programs, which help maintain and define cell types [32-34]

Recent studies indicate that there exists more than 800 known mammalian miRNA genes [35-37] that are conserved throughout evolution with constitutive or spatially and temporally regulated expression. Computational analyses suggest that a single transcript may be regulated by multiple miRNAs [38] and that each miRNA can target tens to hundreds of transcripts [22,31], leading to the conclusion that miRNAs as a whole regulate the expression of at least 30% of human gene transcripts [39]. These discoveries show that miRNAs and their targets are part of complex regulatory networks, for which a number of studies have been performed to reveal the effects of specific miRNAs on temporal and spacial expression of their target genes, and hence the functions of miRNAs. The strategy employed in these studies is to identify the relationship between the expression of a miRNA and its targets by either overexpressing or silencing the miRNA [19,40,41].

Despite all these studies, little is known about miRNA regulation from a genome scale. Accordingly, to gain new insight into miRNA mediated gene regulation in humans, we carried out a series of studies to compare various features of miRNA target genes to that of non-miRNA target genes. These included the difference of gene expression from 79 human tissues [42], mRNA [43] and protein [44] stability, the influence of miRNA binding sites on mRNA degradation, and gene structures. Based on these characteristics of miRNA target genes and by analyzing the relationships between these features, principles of miRNA mediated gene regulation were investigated. To the best of our knowledge, this is the first use of large scale data from different levels to study the mechanism of miRNA regulation in humans, and the findings will therefore help provide new insight into miRNA regulation of human gene expression.

We have found significant differences between miRNA and non-miRNA target genes for a number of characteristics, including higher and broader mRNA expression, faster mRNA decay rate, longer protein half-life, and longer gene structures. We also found that miRNA target genes, other than having miRNA repression, were most likely under more complex regulation than non-miRNA target genes. The results also suggest that higher gene expression and longer length of miRNA target genes may be the consequence of genomic design for regulatory complexity, but it is not the result of lacking "selection for economy", for which highly and broadly expressed genes are compact [45,46]. The higher and broader gene expression but fast mRNA decay rates also suggests that miRNA dampening of the output of preexisting but unwanted transcripts to facilitate transition to new expression programs [32-34] is a general mechanism for miRNA regulation of human gene expression. This can be achieved by enhancing mRNA degradation through an additive effect from multiple miRNA targeting.

## Results

### miRNA target genes have higher and broader expression in human tissues

As a first step to investigate miRNA regulation, we used published gene expression data from the GNF Atlas2 gene expression database from 79 human tissues (gnfAtlas2) [42] to explore both the absolute expression and breadth of expression (See Materials and Methods) differences between non-miRNA and miRNA target genes predicted from the most popular algorithms of TargetScanS [11], PicTar [47], and RNA22 [48]. While both TargetScanS and PicTar predict mammalian miRNA targets based on sequence complementarity, evolutionary conservation, and binding energy, they were reported to have high fidelity for target prediction from biological and informatic validation [11,47]. On the other hand, RNA22, rather than using cross-species conservation as the major component for prediction, employs a pattern-based approach for the identification of miRNA binding sites in the sequence of interest. Therefore, the use of miRNA target genes predicted from RNA22 allows one to avoid potential source of bias due to genes with preferential 3'UTR cross-species conservation.

**Figure 1:**
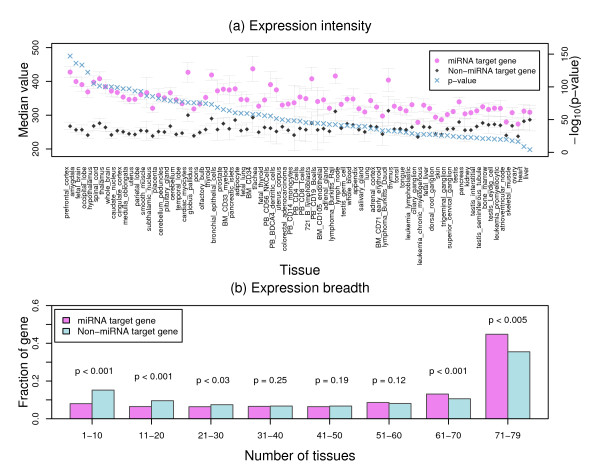
**Expression and expression breadth differences between miRNA and non-miRNA target genes**. (a) Distribution of median expression values from 79 human tissues and *p*-values for the difference between miRNA and non-miRNA target genes. Error bars indicate standard errors. (b) Distribution of the fraction of miRNA and non-miRNA target genes restrictedly expressed in certain number of tissues; *p*-values: statistical differences of median fractions between miRNA and non-miRNA target genes in each bin by Wilcoxon signed rank tests. The results from PicTar predicted miRNA target genes are shown.

For absolute gene expression, we compared the median expression signals of miRNA target genes to those of non-miRNA target genes in ~19620 unique genes within each of the 79 human tissues. The results indicate that miRNA target genes are significantly different from non-miRNA target genes as shown in Figure 1a, where the median expression signals from miRNA target genes derived from PicTar are significantly higher (one-side Wilcoxon rank sum test *p *= 7.8 × 10^-5 ^to 8.5 × 10^-148^) than those from non-miRNA target genes in all 79 human tissues. Similar results were obtained from the other two miRNA target gene sets (Additional file 1). It is interesting to note that the expression differences are especially apparent in brain tissues - a complex tissue with multiple cell types.

For gene expression breadth, we compared the fraction of miRNA target genes restrictively expressed in certain number of tissues to that of non-miRNA target genes. In this analysis, a gene was considered as expressed if its signal exceeded a threshold of 200 arbitrary units according to Su et al [49]. The results indicate that miRNA target genes have wider gene expression breadth compared to that of non-miRNA target genes in 8 ten-tissue bins as shown in Figure 1b, where the fraction of miRNA target genes are, in general, less than the fraction of non-miRNA target genes for genes restrictively expressed in the first 5 tissue bins. However, this is reversed in the last 2 tissue bins where the fraction of miRNA target genes is greater than the fraction of non-miRNA target genes. Comparing the median fraction distribution in each tissue bin, we observed statistically significant differences for the first 3 (one-side Wilcoxon signed rank test *p *< 0.03) and the last 2 (one-side Wilcoxon signed rank test *p *< 0.005) bins, further confirming the characteristics of wide expression breadth for miRNA target genes. Similar results were also obtained from two other miRNA target gene sets (Additional file 2).

We next asked whether any other factors might contribute to these observed gene expression differences between miRNA and non-miRNA target genes. Accordingly, since transcription factors and promoter structures are involved in transcriptional regulation of gene expression, we compared promoters of these two gene groups for GC contents and transcription factor binding sites (TFBSs) (See Materials and Methods). We found that the promoter sequences of miRNA target genes were more GC- and TFBS-enriched when compared to the promoter sequences of non-miRNA target genes. The median GC percentage for promoter sequences of miRNA target genes is ~56%, which is significantly larger (one-side Wilcoxon rank sum test *p *< 10^-200^) than the median percentage of ~52% from non-miRNA target genes. Furthermore, the median TFBS number per promoter is 43 for miRNA target genes, which is also significantly larger (one-side Wilcoxon rank sum test *p *< 10^-200^) than the median TFBS number of 41 from non-miRNA target genes. These results indicate that the gene expression differences between miRNA and non-miRNA target genes may be partially due to transcriptional regulation, a topic that is worthy of further investigation.

### miRNA target genes are less compact

The above findings raise an interesting question why miRNA target genes are highly and widely expressed, as miRNA target genes are down-regulated by miRNAs. Previous reports have shown that highly and broadly expressed genes are shorter in both their intronic and coding sequences than genes expressed at low level or in a few tissues as a results of selection for economy [46,50-52], since transcription and translation are costly. We therefore performed analysis to see if the structures of miRNA target genes were more compact than non-miRNA target genes.

Using the predicted miRNA target gene sets, we compared their structure parameters to those of non-miRNA target genes in ~18870 unique human genes, of which 34%, 34%, and 48% are miRNA target genes from the predictions of TargetScanS, PicTar, and RNA22, respectively. We found that the median lengths of miRNA target genes were significantly longer (one-side Wilcoxon rank sum test *p *< 10^-250^) than those of non-miRNA target genes in all parts of gene structures (Table 1). These findings were consistent in all comparisons from the three miRNA target gene sets. The top 2 largest differences were observed from 3'UTRs and introns, two gene structures playing important roles in gene regulation and organism complexity [53]. While the median 3'UTR length of miRNA target genes was between 1.96 and 2.62 fold of non-miRNA target genes, the median intron length had more than 1.76 fold difference between miRNA and non-miRNA target genes. The smallest difference was observed in 5' untranslated regions (5'UTRs), whose median length of miRNA target genes was still 15% longer than that of non-miRNA target genes.

**Table 1:** miRNA target genes are less compact

	miRNA target genes	Non-miRNA target genes	Fold difference	Rank order
**TargetScanS**	n = 6444	n = 12428		
Coding sequences length	2009 ± 26 (1512)	1575 ± 14 (1182)	1.28 1.28	6
3'UTR length	1730 ± 19 (1303)	1251 ± 11 (498)	1.38 2.62	1
5'UTR length	274 ± 3 (214)	197 ± 2 (131)	1.39 1.63	3
mRNA length	3989 ± 33 (3460)	2705 ± 19 (2161)	1.48 1.60	4
Total intron length	83699 ± 1890 (35802)	42275 ± 859 (14315)	1.98 2.50	2
Number of introns	11.6 ± 0.14 (9)	8.5 ± 0.01 (6)	1.37 1.50	5
				
**PicTar**	n = 6545	n = 12327		
Coding sequences length	1771 ± 22 (1346)	1264 ± 13 (873)	1.40 1.54	4
3'UTR length	1688 ± 18 (1286)	965 ± 12 (497)	1.75 2.59	1
5'UTR length	271 ± 3 (214)	198 ± 2 (131)	1.37 1.63	3
mRNA length	3871 ± 32 (3363)	2757 ± 20 (2180)	1.40 1.54	4
Total intron length	80741 ± 1792 (33818)	43507 ± 910 (14564)	1.86 2.32	2
Number of introns	11.1 ± 0.14 (9)	8.5 ± 0.09 (6)	1.28 1.50	5
				
**RNA22**	n = 9055	n = 9817		
Coding sequences length	1833 ± 20 (1398)	1622 ± 16 (1191)	1.13 1.17	5
3'UTR length	1415 ± 14 (976)	1032 ± 14 (499)	1.41 1.96	1
5'UTR length	231 ± 2 (171)	216 ± 3 (149)	1.07 1.15	6
mRNA length	3455 ± 26 (2898)	2856 ± 23 (2240)	1.21 1.29	4
Total intron length	61231 ± 1201 (25368)	51983 ± 1250 (14451)	1.18 1.76	2
Number of introns	11.6 ± 0.1 (8)	8.8 ± 0.1 (6)	1.32 1.33	3

Comparison of the structure parameters of non-miRNA target genes with those of miRNA target genes predicted from TargetScans, PicTar, and RNA22. For each gene structure parameter the first line gives the average value ± s.e.m, and the 2^nd ^line the median values in brackets. n: the number of genes in each category. The median lengths of miRNA target genes were significantly longer (one-side Wilcoxon rank sum test *p *< 10^-250^) than those of non-miRNA target genes in all parts of gene structures.

The result that miRNA target genes have longer 3'UTR sequences is not surprising, as miRNAs perform their regulation roles mainly by pairing with 3'UTR of target genes. However, the finding that other gene structures of miRNA target genes are also longer is contradicted to the "selection for economy", for which highly and broadly expressed genes are compact [45,46]. Previous studies indicated that longer gene structures were most likely linked to gene regulation, such as splicing regulation and chromatin-mediated gene suppression from introns [46,54]. The result therefore indicates that miRNA target genes, other than having miRNA repression, are subject to more complex regulation [32-34,52,54].

### miRNA target transcripts are less stable but not their protein products

It is widely accepted that miRNAs mediate gene regulation by reducing the stability of their target transcripts. Previous studies from manipulating individual miRNAs revealed that introducing an alien miRNA into human HeLa cells down-regulated a large number of mRNAs [19] and that inhibiting miR-122 function in mouse liver led to the increase of stability for hundreds of mRNAs [41]. To investigate if mRNA stability of miRNA target genes differs from that of non-miRNA target genes *on the genome scale*, we compared the mRNA decay rates between these two gene groups using data from Yang *et al*. [43], which contains mRNA decay rates for ~5550 human transcripts, representing ~4250 unique genes. Out of the 4250 genes, 2148, 2020, and 2248 were mapped to miRNA target genes predicted from TargetScanS, PicTar, and RNA22, respectively.

**Figure 2:**
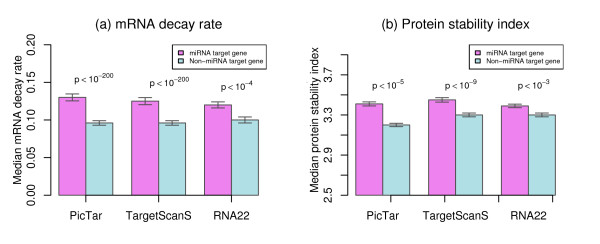
**Comparison of mRNA decay rate and protein stability between miRNA and non-miRNA target genes**. (a) miRNA target genes on average have higher mRNA decay rates than non-miRNA target genes. (b) miRNA target genes on average have longer protein half-lives than non-miRNA target genes. Target genes predicted by three algorithms of PicTar, TargetScanS, and RNA22 are shown. Error bars indicate standard errors. *p: p*-values from one-side Wilcoxon rank sum tests for the median value differences between miRNA and non-miRNA target genes.

The results indicate that the median mRNA decay rates for miRNA target genes (0.12 to 0.13 h^-1^) are between 1.2 and 1.35 fold of that obtained from non-miRNA target genes (0.096 to 0.1 h^-1^). This observation of higher mRNA decay rate for miRNA target genes is true for genes predicted from all three algorithms as shown in Figure 2a. Statistical analyses using one-side Wilcoxon rank sum tests revealed significant differences of mRNA decay rates between miRNA and non-miRNA target genes, with *p*-values < 10^-200^for the comparisons using miRNA target genes predicted from TargetScanS and PicTar and *p*-values < 10^-4 ^for the comparison using miRNA target genes predicted from RNA22. These findings, which are in agreement with previous reports from individual miRNA studies [19,40,41], suggest that miRNAs enhance the down-regulation of target mRNAs through increasing their decay rate.

As an extension of selection for economy for genes, one would expect that genes with a lower mRNA decay rate should be more stable for their protein products. Therefore, we asked if a similar difference existed for their protein products, although protein stability is not likely under miRNA regulation. To address this question, we compared protein stability of miRNA target genes to that of non-miRNA target genes using data from Yen *et al*. [44], which contains half-life protein stability measures represented by protein stability indices for ~6500 unique genes. Out of the 6500 genes, we were able to map 2417, 2502, and 3366 to miRNA target genes predicted from TargetScanS, PicTar, and RNA22, respectively. Contrary to the selection for economy for genes, the results indicate that proteins of miRNA target genes are more stable than those of non-miRNA target genes as shown in Figure 2b, where the median protein stability indices from miRNA target genes are larger than those from non-miRNA target genes. Further statistical analyses using one-side Wilcoxon rank sum tests revealed significant differences between miRNA and non-miRNA target genes, with *p*-values of 6.4 × 10^-10^, 6.3 × 10^-6^, and 9 × 10^-4 ^for the comparisons using miRNA target genes predicted from TargetScanS, and PicTar, and RNA22, respectively.

### miRNA target gene expression is correlated with mRNA and protein stability

The above discovered characteristics of higher and broader mRNA expression, faster mRNA decay rate, and longer protein half life for miRNA target genes raise an important question as to whether or not they are related to each other. If these characteristics associate with miRNA target genes and are dependent on each other, then they are expected to correlate from the same set of miRNA target genes. This should be especially true for mRNA expression and decay rate, as both are related to miRNA targets. To address this question, we first mapped miRNA target genes from mRNA expression, mRNA decay rate, and protein stability index datasets to each other. We then computed the correlation using the paired values from the overlapping genes.

**Figure 3:**
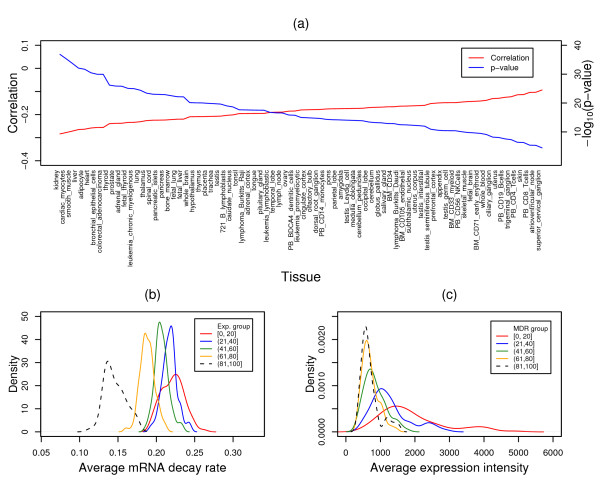
**Correlation between mRNA expression and decay rate**. (a) Spearman's rank correlation rho between gene expression from each of the 79 human tissues and mRNA decay rates, and corresponding *p*-values (-log_10_(p-values)) for the correlation coefficients. (b) Distribution of the average mRNA decay rates, which were obtained from comparing gene expression in each of the 79 human tissues, for 5 mRNA expression groups with increasing expression values from the group [1-20] to the group (81,100]. (c) Distribution of the average mRNA expression values in the 79 human tissues for 5 mRNA decay rate groups with increasing mRNA decay rate from the group [1-20] to the group (81,100]. Exp: mRNA expression; MDR: mRNA decay rate.

The findings indicate that mRNA decay rate and expression are negatively correlated. Figure 3a shows the results from PicTar predicted miRNA target genes, whose expression values from all 79 human tissues are significantly and inversely correlated with mRNA decay rate (Spearman's rank correlation rho: between -0.1 and -0.28; *p*: between 3.4 × 10^-5 ^and 14 × 10^-37^). To further confirm our findings, we employed the rank test to directly assess the relationship between mRNA expression and decay rate. In agreement with the correlation results, as the intensity of mRNA increases, the average level of mRNA decay rate decreases as shown in Figure 3b, where the distribution of the average mRNA decay rate for the 5 expression groups is depicted. Statistical analyses showed that the average mRNA decay rates in high expression groups were significantly smaller than those in low expression groups (one-side Wilcoxon signed rank test *p *< 10^-9^). This observation was also true for the mRNA decay rate ranked analysis as shown in Figure 3c, where the average expression from 79 human tissues decreases along with the increasing of mRNA decay rate, with significant expression differences (one-side Wilcoxon signed rank test *p *< 10^-13^) between low, medium, and high mRNA decay rate groups.

**Figure 4:**
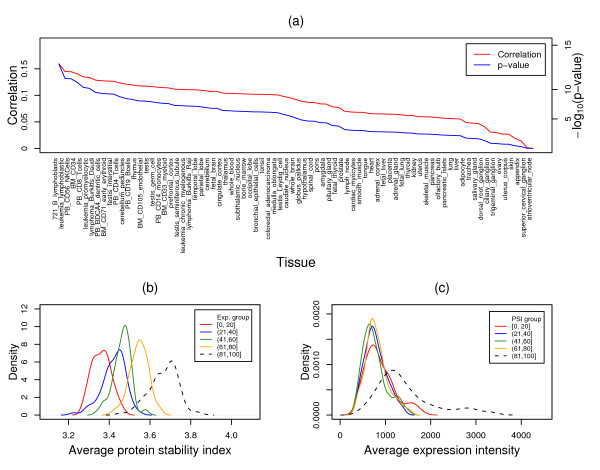
**Correlation between gene expression and protein stability**. (a) Spearman's rank correlation rho between gene expression from each of the 79 human tissues and protein stability, and corresponding *p*-values (-log_10_(p-values)) for the correlation coefficients. (b) Distribution of the average protein stability indices, which were obtained from comparing gene expression in each of the 79 human tissues, for 5 mRNA expression groups with increasing expression values from the group [1,20] to the group (81,100]. (c) Distribution of the average mRNA expression values in the 79 human tissues for 5 protein stability groups with increasing protein stability index from the group [1,20] to the group (81,100]. Exp: mRNA expression; PSI: protein stability. Index.

Unlike the mRNA decay rate, protein stability was in general positively correlated with mRNA expression as shown in Figure 4a, where 78 of 79 tissues display positive correlations, with 71 tissues having significant correlations (Spearman's rank correlation test *p *between 4.5 × 10^-2 ^and 5.5 × 10^-15^). Further rank tests revealed that as the intensity of mRNA increases, the average protein stability also increases as shown in Figure 4b, where the distribution of the average protein stability for 5 expression groups is depicted. Statistical analyses showed that protein stability between the low, medium, and high expression groups were significantly different (one-side Wilcoxon signed rank test *p *< 10^-9^). Similar results was also observed for protein stability ranked analysis as shown in Figure 4c, where the average expression from 79 human tissues increases along with the increasing of protein stability, with significant expression differences (one-side Wilcoxon signed rank test *p *< 10^-14^) between medium and high protein stability groups.

Although we observed negative correlations between mRNA decay rate and protein stability (R: between -0.027 and -0.09), these correlations are not statistically significant across all three miRNA target gene sets (Spearman's rank correlation *p*: between 0.4 and 7 × 10^-4^), suggesting that mRNA decay rate and protein stability are most likely independent. Similar correlation results between mRNA decay rate, mRNA expression, and protein stability were obtained from TargetScanS and RNA22 predicted miRNA target genes (Additional files 3, 4, 5, and 6).

Taken together, the significant and inverse correlation between mRNA expression and mRNA stability but positive correlation to protein stability and the higher and broader gene expression but fast mRNA decay rates for miRNA target genes suggest that miRNA dampening the output of preexisting but unwanted transcripts to facilitate transition to new expression programs [32-34] is a general mechanism for miRNA regulation of gene expression.

### miRNA binding sites have an additive effect on mRNA stability

As a first step to understand how miRNAs facilitate transition of miRNA target genes to new expression programs, we sought to determine if the number of miRNA binding sites had any effect on the level of mRNA degradation. Accordingly, we performed analyses to investigate the influence of the number of miRNA binding sites on mRNA stability by examining the relationship between the number of miRNA binding sites and the level of mRNA decay rate in miRNA target genes.

**Figure 5:**
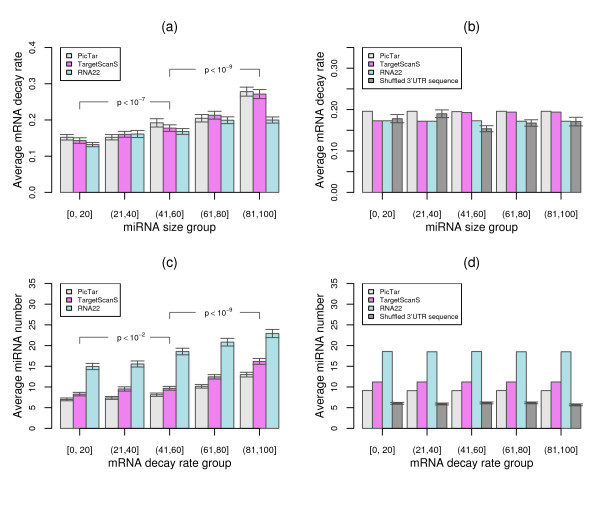
**Relationship between the number of miRNA binding sites and mRNA decay rate**. (a) Variation of mRNA decay rates for 5 miRNA binding site groups with increasing number of binding sites. The degree of the increasing trend for mRNA decay rates along with the increasing number of miRNA binding sites was estimated by Wilcoxon rank sum tests and depicted in p-values. (b) The expected variation of mRNA decay rates from both permutation tests and shuffled 3'UTR sequences for 5 miRNA binding site groups with increasing number of binding sites. (c) Variation of miRNA binding site number for 5 mRNA decay rate groups with increasing decay rates. The degree of the increasing trend for miRNA binding site number along with the increasing mRNA decay rates was estimated by Wilcoxon rank sum tests and depicted in p-values. (d) The expected variation of miRNA binding site number from both permutation tests and shuffled 3'UTR sequences for 5 mRNA decay rate groups with increasing decay rates.

Using these 2148, 2020, and 2248 miRNA target genes that had mRNA decay rate data as described above, we first performed correlation analyses to see whether the number of miRNA binding sites and mRNA decay rate were related to each other. The results indicated that the number of miRNA binding sites was positively and significantly (one-side Spearman's rank correlation *p *< 10^-15^) correlated with mRNA decay rate in all three miRNA target gene sets with correlation coefficients between 0.17 and 0.22. In an effort to extend our analyses for direct comparison, we employed rank tests to assess the relationship between the number of miRNA binding sites and the level of mRNA decay rate based on the rank order either from miRNA decay rate or from the number of miRNA binding sites (See Materials and Methods). Figure 5a shows that as the number of miRNA binding sites increases, the level of mRNA decay rate steadily increases. Statistical analyses showed that the mRNA decay rate between the low, medium, and high miRNA number groups was significantly different (one-side Wilcoxon rank sum test *p*: 3.6 × 10^-8 ^to 7.8 × 10^-12^), confirming the changing trends of mRNA decay rate along with the number of miRNA binding sites. This observation is also true for the mRNA decay rate ranked analysis as shown in Figure 5c, where the average number of miRNA binding sites increases along with the increasing mRNA decay rate, with significant differences for the number of miRNA binding sites (one-side Wilcoxon rank sum test *p*: 7 × 10^-3 ^to 6 × 10^-13^) between low, medium, and high mRNA decay rate groups.

We performed the following 4 analyses to verify that the observed additive effect of miRNA binding sites on mRNA decay rates was indeed a property for miRNA regulation. In the first analysis, we assessed the robustness of the changing trends for the additive effect of miRNA binding sites on mRNA decay rate by permutation tests (See Methods). No changing trend was obtained from any of 10,000 random datasets as shown in Figure 5b and Figure 5d, where the mRNA decay rate or miRNA binding sites from the 10,000 random datasets were displayed along with the corresponding miRNA binding site or mRNA decay rate groups.

We next performed analysis to reveal whether the additive effect was contributed from biologically relevant miRNA binding sites, or rather if it came from other factors. Accordingly, we first created 3 random datasets by shuffling the 3'UTR sequences of the 2248 RNA22 predicted miRNA target genes which had mRNA decay rate data. We then used the RNA22 algorithm to predict miRNA binding sites for individual genes in these random datasets (See Materials and Methods). We subsequently employed the rank test to assess the relationship between the number of miRNA binding sites from the 3'UTR sequences of these random datasets and the level of mRNA decay rate in corresponding genes. Again, no positive correlations were observed between miRNA decay rate and the number of miRNA binding sites from any of the three random datasets as also shown in Figure 5b and Figure 5d, where the results from the complete nucleotide mixing dataset are depicted.

**Figure 6:**
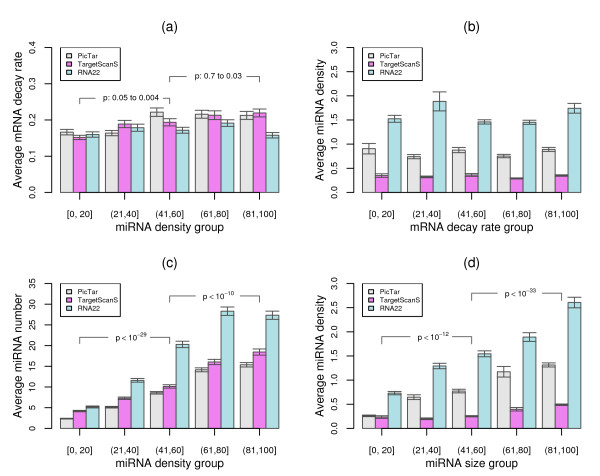
**Relationship between miRNA density, mRNA decay rate, and the number of miRNA binding sites**. (a) Variation of mRNA decay rates for 5 miRNA density groups with increasing miRNA density. The degree of the increasing trend for mRNA decay rates along with the increasing miRNA density was estimated by Wilcoxon rank sum tests and depicted in p-values. (b) Variation of miRNA density for 5 mRNA decay rate groups with increasing decay rate values. (c) Variation of miRNA binding site number for 5 miRNA density groups with increasing density values. The degree of the increasing trend for miRNA binding site number along with the increasing miRNA density was estimated by Wilcoxon rank sum tests and depicted in p-values. (d) Variation of miRNA density for 5 miRNA binding site groups with increasing number of binding sites. The degree of the increasing trend for miRNA density along with the increasing number of miRNA binding sites was estimated by Wilcoxon rank sum tests and depicted in p-values.

We also verified that the additive effect was mainly contributed from the number of miRNA binding sites but not from miRNA density on 3'UTR sequences. In this analysis, we first computed miRNA density, which was represented as the number of miRNA binding sites per 100 nucleotides on 3'UTR sequences, and then employed the rank test to assess the relationship between miRNA density and the level of mRNA decay rate. We found that although the level of mRNA decay rate displayed changes along with the increasing miRNA density (Figure 6a), these changes were neither consistent in different miRNA target gene sets nor statistically significant between miRNA density groups for most of the comparisons. Furthermore, no trend was observed for miRNA density between different mRNA decay rate groups (Figure 6b), suggesting that miRNA density has little effect on mRNA degradation. We however found that miRNA density was dependent on the number of miRNA binding sites on target genes (Figure 6c and Figure 6d). Therefore, the influence of miRNA density, if any, on mRNA stability is most likely due to the number of miRNA binding sites.

**Figure 7:**
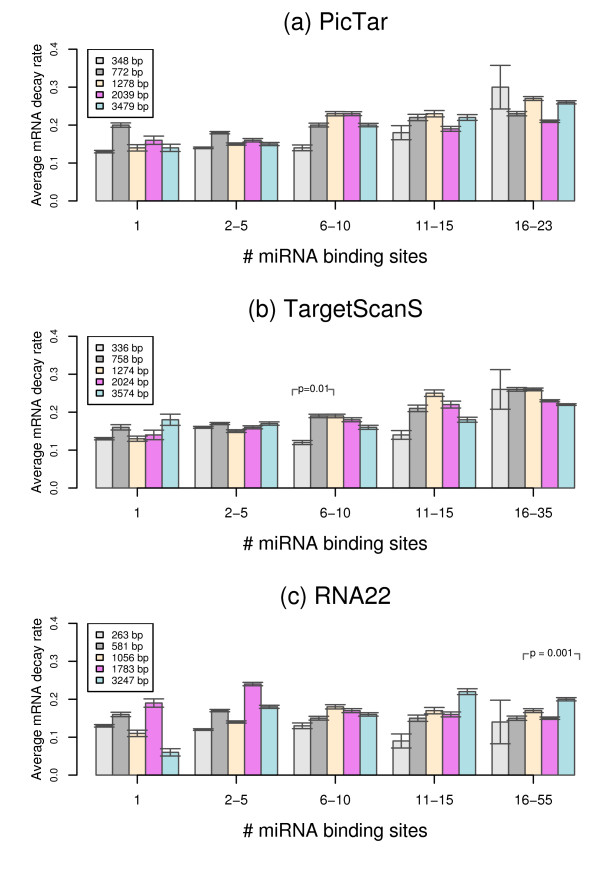
**Relationship between the length of 3'UTR and mRNA decay rate**. Variation of mRNA decay rates of different 3'UTR length groups for miRNA target genes with different miRNA target sites predicted from PicTar (a), TargetScanS (b), and RNA22 (c). The median length of each 3'UTR length group is shown in the legend boxes. The degree of the mRNA decay rate differences between the low, median, and high 3'UTR length groups in each miRNA binding site groups was estimated by Wilcoxon rank sum tests and the significant ones are depicted in *p*-values.

We finally performed analysis to see if mRNA decay rates were related to the length of 3'UTR sequences. In this analysis, we first grouped miRNA target genes based on their 3'UTR length, we then compared the decay rates of miRNA target genes with similar number of miRNA binding sites across different 3'UTR length groups. The results indicated that no correlation existed between mRNA decay rates and 3'UTR length for genes with either a few or many miRNA target sites across 5 different 3' UTR length groups as shown in Figure 7, where no consistent trend is observed for mRNA decay rates between different 3'UTR length groups. Statistical analyses (two-side Wilcoxon rank sum test) showed that mRNA decay rates between the low, medium, and high 3'UTR length groups were, in general, not significantly different except 1 comparison from miRNA target genes predicted from TargetScanS (Figure 7b) and 1 comparison from miRNA target genes predicted from RNA22 (Figure 7c). These results indicated that the 3'UTR length itself had little effect on mRNA degradation, further exemplifying the additive effect of miRNA binding sites on mRNA stability.

## Discussion

miRNAs regulate gene expression post-transcriptionally by base-pairing to target mRNAs mainly in 3'UTRs. It has been demonstrated that genes preferentially coexpressed with miRNAs have evolved to avoid targeting by that miRNA, a phenomenon referred to as antitargets. Genes of this kind are able to escape from miRNA targeting by site depletion [33], for which genes have either short UTRs or low target site densities. One of the examples to avoid miRNA regulation is the housekeeping genes which have not only shorter 3'UTRs than other transcripts but also lower target site density. Further evidence for antitargets came from correlation study between "seed" sequences and the expression of several highly tissue-specific human miRNAs, which specifically down-regulate a large number of mRNA targets. The down-regulation was correlated to the presence of miRNA seed sequence in 3'UTRs of target mRNAs [55].

Since miRNAs can down-regulate target mRNA, one would expect that miRNA target genes should be expressed at lower levels than non-miRNA target genes. We however found significantly higher mRNA expression for miRNA target genes from different human tissues, when compared to non-miRNA target genes. The high mRNA expression of miRNA target genes might be partly due to their promoter sequences, which generally have more TFBSs and are enriched in GC nucleotides, a topic that is worthy of further investigating. On the other hand, we observed that miRNA target genes had high mRNA decay rates but were more stable for their protein products. Further analyses indicated that mRNA expression of miRNA target genes was significantly and inversely correlated with mRNA stability but positively correlated with protein stability. The highly expressed mRNA from miRNA target genes is subjected to more rapid degradation but the protein products are more stable. These observations raise an interesting question as to why mRNA of miRNA target genes are highly transcripted and then degraded before reaching the final protein products, as the transcriptional process is costly.

One explanation for the above phenomenon is that mRNA of miRNA target genes are generally subjected to tuning, which is one way that miRNAs regulate their targets based on a micromanager model [56]. In this model, the miRNA targets were classified into a few categories, with one class comprising of switch targets with multiple target sites presenting in the 3'UTR. With the onset of new developmental stage or other cellular changes these miRNA targets take advantage of the miRNAs to dampen gene products to inconsequential levels so that new expression programs can be achieved. This post-transcriptional control could be more responsive than transcriptional control in terms of both speed and reversibility. One of the examples came from the study for zebrafish, in which miR-430 directly regulated several hundred targets by promoting deadenylation and clearance of mRNAs. Most of these mRNAs were maternally expressed and accumulated in the absence of miR-430 but were degraded during early embryogenesis [18]. In agreement with others' discoveries from individual genes, our findings from genome scale analyses on the characteristics of miRNA target genes also suggest that in human miRNA dampening of the preexisting transcript outputs could be a general mechanism for miRNA regulation. Furthermore, miRNA target genes are most likely under more complex regulation than non-miRNA target genes.

Supporting the complex regulation notion, miRNA target genes in humans are significantly longer than non-miRNA target genes in various parts of gene structures. The longer gene structures but higher gene expression for miRNA target genes seems wasteful based on "selection for economy" for genes [45,46,51], for which the highly and broadly expressed genes are shorter both in their intronic and coding sequences than genes expressed in a narrow and low fashion, as transcription and translation are energetically costly. Considering the enhanced mRNA decay rate for miRNA target genes, the paradox against the selection for economy is even more apparent. The results from this study therefore suggest that evolution may not necessarily optimize efficiency but impose specific roles for miRNA target genes such as more subtle and complex gene regulation based on a "genome design" hypothesis [52]. This hypothesis suggests that the length of gene structures is determined by their function and that longer gene structures are involved in more complex regulation such as chromatin-mediated suppression of gene expression.

In this study, we observed significant correlations between mRNA decay rate and the number of miRNA binding sites for miRNA target genes. We found that miRNA binding sites had an additive effect on mRNA decay rate, for which genes with more miRNA binding sites displayed higher mRNA decay rate. These additive effects come mainly from different miRNAs, since only a small fraction of all predicted targets contain more than one miRNA binding site for any single miRNA [34]. While the number of miRNA binding sites for miRNA target genes used in this study ranges from 1 to 173, with average number > 8, more than 82% of genes have 2 or more miRNA binding sites, indicating that majority of miRNA target genes are under multiple miRNA regulation. It is suggested that the main reason for having multiple miRNA targeting on individual genes is that these genes can be regulated in a variety of conditions such as developmental timing and within various tissues. Results from this study revealed that multiple targeting on a gene could also act together to provide enhanced mRNA decay rate, and hence stronger gene repression, which could contribute to facilitate the transition of miRNA target genes to new expression programs.

## Conclusion

To gain new insight into miRNA regulation in humans, we have used large scale data and carried out a series of studies to compare various features of miRNA target genes to that of non-miRNA target genes. We have demonstrated that miRNA and non-miRNA target genes are significantly different for a number of characteristics, including higher and broader mRNA expression, faster mRNA decay rate, longer protein half-life, and longer gene structures. We have found that miRNA target genes, other than having miRNA repression, are most likely under more complex regulation than non-miRNA target genes. We have also revealed that miRNA dampening the output of preexisting but unwanted transcripts to facilitate transition to new expression programs is a general mechanism for miRNA regulation of human gene expression. This could be achieved by enhancing mRNA degradation through an additive effect from multiple miRNA targeting.

## Methods

### Compilation of human and miRNA target genes

Human genes (hg18 March 2006 assembly) with Reference Sequences (RefSeqs) and their corresponding structure parameters, including the number of introns, the length of coding sequences (CDSs), introns, 3'UTRs, and 5'UTRs, were extracted from UCSC Genome browser http://genome.ucsc.edu/. To allow comparisons between different data sources, the RefSeqs IDs were mapped to the corresponding Gene IDs from NCBI http://www.ncbi.nlm.nih.gov/ using gene2refseq. This process resulted in ~27600 RefSeqs, which represent ~18870 unique genes, with structure parameters.

Three miRNA target gene datasets predicted from algorithms of PicTar [47], TargetScanS [11], and RNA22 [48] were used. miRNA target genes from PicTar and TargetScanS were obtained from miRGen database [57], which contains 7034 and 6995 unique target genes for PicTar and TargetScanS, respectively. For PicTar, the result based on conservation in human, chimp, mouse, and rat was chosen. For TargetScanS, the predictions using positions on UTRs (without gaps) and 4-way UTR multiple sequence alignments were used. miRNA target genes predicted from RNA22 were obtained from RNA22 web server http://cbcsrv.watson.ibm.com/rna22.html, which contains ~21650 Ensemble transcripts of miRNA targets. To make them comparable to other data sources, these Ensemble transcripts, which represent ~9240 unique genes, were mapped to Gene IDs based on Biomart http://www.biomart.org/. The number of miRNA target sites from multiple miRNAs for individual genes was also computed for each dataset. For a given gene with multiple predicted transcripts from RNA22, the transcript with the most miRNA binding sites was chosen. We also employed miRNA target genes common between TargetScanS and PicTar for all analyses performed in this study. Results (data not shown) similar to the miRNA target genes predicted from the three individual algorithms were obtained.

For each miRNA target gene dataset, the ~18870 unique human genes were first separated into two groups of miRNA and non-miRNA target genes and their structure parameters were then compared. In the case of a gene with alternative-splicing variants the one having the largest gene structure parameter was used. Significant differences for the median values between miRNA and non-miRNA target genes were determined by Wilcoxon rank sum test.

### Human gene expression data and expression comparison

The GNF Atlas2 gene expression database (gnfAtlas2) [49] was used for the gene expression comparisons between miRNA and non-miRNA target genes. This dataset, which contains ~16920 unique genes whose mRNA expression was determined in 79 human tissues, cell types, and cancer lines has been widely used for various gene expression studies. The expression signals for a given gene with different RefSeq IDs in the same tissue were first averaged. miRNA target genes derived from TargetScanS, PicTar, and RNA22 were then mapped to the ~16920 unique genes based on their gene IDs. Out of 5166 to 9235 miRNA target genes, 6245 from TargetScanS, 6300 from PicTar, and 8276 from RNA22 were in the expression data gene list. The median expression levels between miRNA and non-miRNA target genes were compared, and the statistical significances were evaluated using Wilcoxon rank sum test.

For the comparison of gene expression breadth, expressed miRNA and non-miRNA target genes were first extracted from the dataset. A gene is considered to be expressed if its expression intensity exceeds a threshold of 200 arbitrary units according to Su et al [49]. The resulting miRNA and non-miRNA target genes were then separated into different bins based on their expression breadth across 79 tissues. A total of 8 bins were defined with increment of 10 tissues for the first 7 bins and increment of 9 tissues for the 8^th ^bin. Therefore, each bin contains genes expressed in its specified tissue numbers. For example, the first bin contains genes expressed in 1 to 10 tissues and the 8^th ^bin contains genes expressed in 71 to 79 tissues. Comparisons between miRNA and non-miRNA target genes were performed within each bin, in which each of the 10 (9) fractions of miRNA target genes out of all expressed miRNA target genes was compared to that of non-miRNA target genes. The statistical significance between miRNA and non-miRNA target genes in each bin was determined by Wilcoxon signed rank test, which evaluates the difference for the 10 paired fractions of miRNA and non-miRNA target genes.

### GC content and TFBSs comparisons

Promoter sequences within 1-kb upstream of transcriptional starting sites for both miRNA and non-miRNA target genes were extracted from the UCSC Genome browser (hg18 March 2006 assembly). The percentage of GC nucleotides in each promoter sequence was computed using an in-house developed PERL script, and the differences of the median values between miRNA and non-miRNA target genes were evaluated by Wilcoxon rank sum test. For TFBS comparison, the Match^® ^program, for which the profile parameter was set to "minimize the false positives", was employed to conduct searches for TFBSs using 214 non-redundant vertebrate position weight matrices from the professional TRANSFAC11.4 database [58]. The number of TFBSs on each promoter sequence was computed using an in-house developed PERL script, and the differences of the median values between miRNA and non-miRNA target genes were evaluated by Wilcoxon rank sum test.

### Compilation of mRNA decay rate and protein stability data

mRNA decay rate data were obtained from Yang *et al*. [43], which contains mRNA decay rates for ~5550 human transcripts. To allow comparisons between different data sources, the Accession IDs were first mapped to the corresponding Gene IDs from NCBI http://www.ncbi.nlm.nih.gov/ using gene2refseq. The mRNA decay rate for a given gene with different Accession IDs was then averaged, resulting in ~4250 unique genes with mRNA decay rate. Protein stability data were obtained from Yen *et al*. [44], which contains half-life protein stability measures represented as protein stability index for ~6500 unique genes. For genes with multiple tests, the values of protein stability indices were averaged.

### Rank tests

Rank tests were employed for a few analyses, including miRNA binding sites vs. mRNA decay rates, miRNA binding sites vs. protein stability, miRNA density vs. mRNA decay rates, miRNA density vs. miRNA binding sites, mRNA decay rates vs. mRNA expression, and protein stability vs. mRNA expression. Taking the miRNA binding sites vs. mRNA decay rates as an example, genes were first ranked by either the number of miRNA binding sites or mRNA decay rates, resulting in 2 datasets with either ordered number of miRNA binding sites or ordered mRNA decay rates. Genes in each of the two resulting datasets were then separated into 5 equal number groups, which were represented as [1,20], (21,40], (41,60], (61,80], and (81,100] in standard interval notation, with increasing values. Comparison analyses for the paired values of miRNA binding sites and mRNA decay rates were performed within each of the 5 gene groups.

### Significance tests

To assess the robustness of the trends for the additive effect of miRNA binding sites on mRNA decay rate, permutation test was first performed to create 10,000 datasets by randomizing samples of miRNA binding sites and mRNA decay rate from genes of the test datasets. Each of the random datasets was then subjected to rank test as described above. Wilcoxon rank sum tests were subsequently employed to determine the statistical difference between [1,20] and (41,60] groups as well as between (41,60] and (81,100] groups in each random dataset. The resulting 10,000 p-values were compared to that from test dataset to estimate the robustness of the trends for the observed additive effect of miRNA binding sites on mRNA decay rate.

We also performed analysis to test whether the additive effects of miRNA binding sites on mRNA decay rate were contributed from biologically relevant miRNA binding sites. For this analysis, we first created 3 random 3'UTR sequence datasets, each having the same number of genes and nucleotide contents as the test dataset. Specifically, we shuffled the 3'UTR sequences within each of the 2248 RNA22 predicted miRNA target genes which have mRNA decay rate data by either keeping dinucleotide together or mixing completely. The resulting datasets (2 from keeping dinucleotide together and 1 from complete mixing) were then subjected to the prediction of miRNA binding sites through the RNA22 web server http://cbcsrv.watson.ibm.com/rna22.html. To make the predictions comparable to those of the test dataset, we compiled and used the same list of miRNAs based on the test dataset and set up the parameters for RNA22 algorithm to be 0 for "maximum number of allowed UN-paired bases", 6 for "in seed/nucleus", 14 for "minimum number of paired-up bases in heteroduplex", and -25 for "maximum folding energy for heteroduplex".

## Supplementary Material

Additional file 1 **Supplemental Figure 1**. Shows the expression differences between non-miRNA and miRNA target genes predicted from TargetScanS and RNA22. Click here for file

Additional file 2 **Supplemental Figure 2**. Shows the expression breadth differences between non-miRNA and miRNA target genes predicted from TargetScanS and RNA22. Click here for file

Additional file 3 **Supplemental figure 3**. Shows the correlation between mRNA expression and decay rate for miRNA target genes predicted from TargetScanS. Click here for file

Additional file 4 Shows the correlation between mRNA expression and decay rate for miRNA target genes predicted from RNA22. Click here for file

Additional file 5 Shows the correlation between gene expression and protein stability for miRNA target genes predicted from TargetScanS. Click here for file

Additional file 6 Shows the correlation between gene expression and protein stability for miRNA target genes predicted from RNA22. Click here for file
