# Supplementary material for: Insight into microRNA regulation by analyzing the characteristics of their targets in humans
Source: BMC Genomics. 2009 Dec 10;10:594. doi: 10.1186/1471-2164-10-594 (PMC2799441; doi:10.1186/1471-2164-10-594)
Supplement: Additional file 1 — Supplemental Figure 1. Shows the expression differences between non-miRNA and miRNA target genes predicted from TargetScanS and RNA22. [file 1471-2164-10-594-S1.PDF]

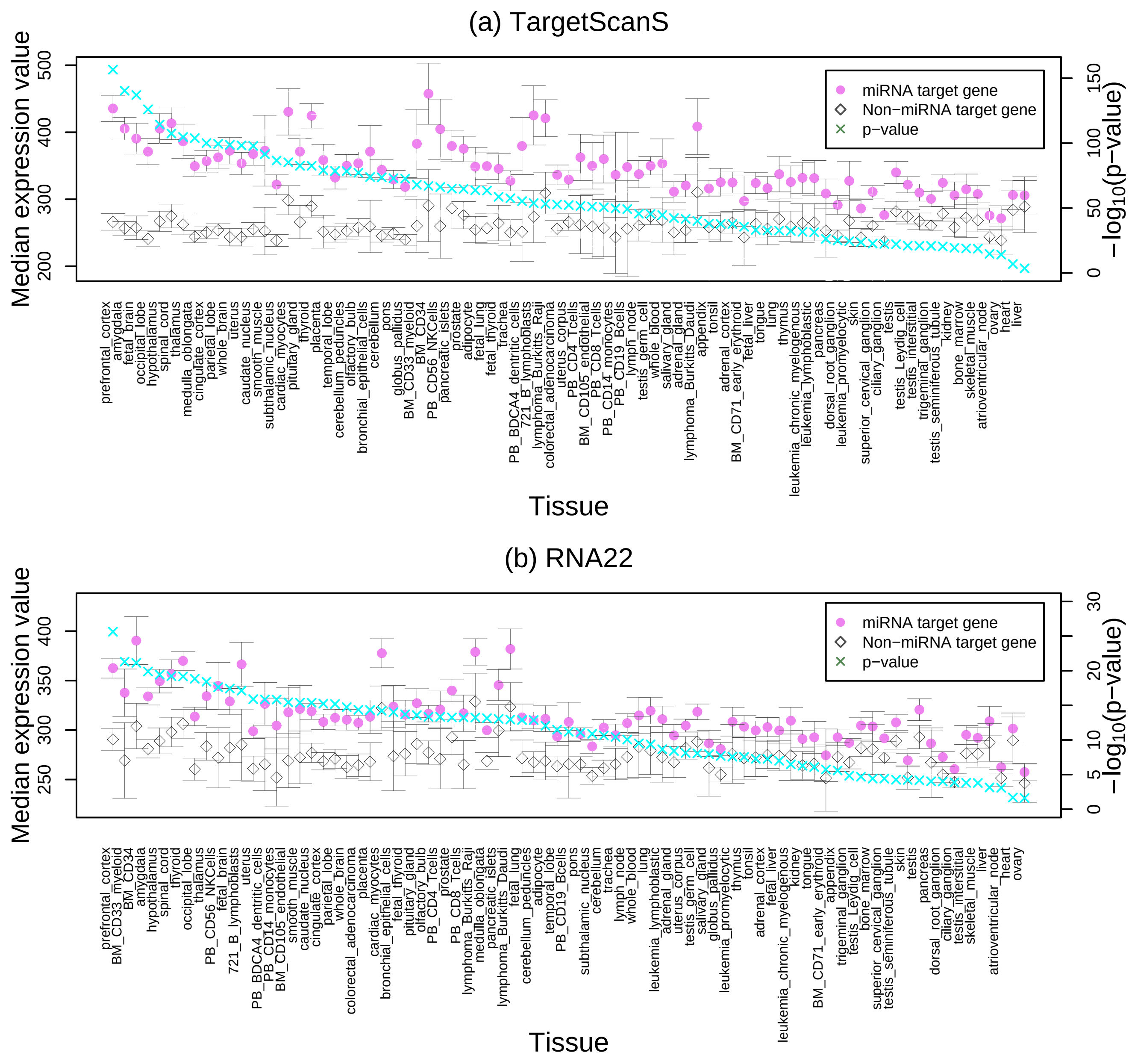

Figure S1. Expression differences between non-miRNA and miRNA target genes predicted from TargetScanS and RNA22. Distribution of median expression values from 79 human tissues and p-values for the difference between non-miRNA and miRNA target genes predicted from TargetScanS (a) and RNA22 (b). Error bars indicate standard errors.
