# Supplementary material for: Insight into microRNA regulation by analyzing the characteristics of their targets in humans
Source: BMC Genomics. 2009 Dec 10;10:594. doi: 10.1186/1471-2164-10-594 (PMC2799441; doi:10.1186/1471-2164-10-594)
Supplement: Additional file 2 — Supplemental Figure 2. Shows the expression breadth differences between non-miRNA and miRNA target genes predicted from TargetScanS and RNA22. [file 1471-2164-10-594-S2.PDF]

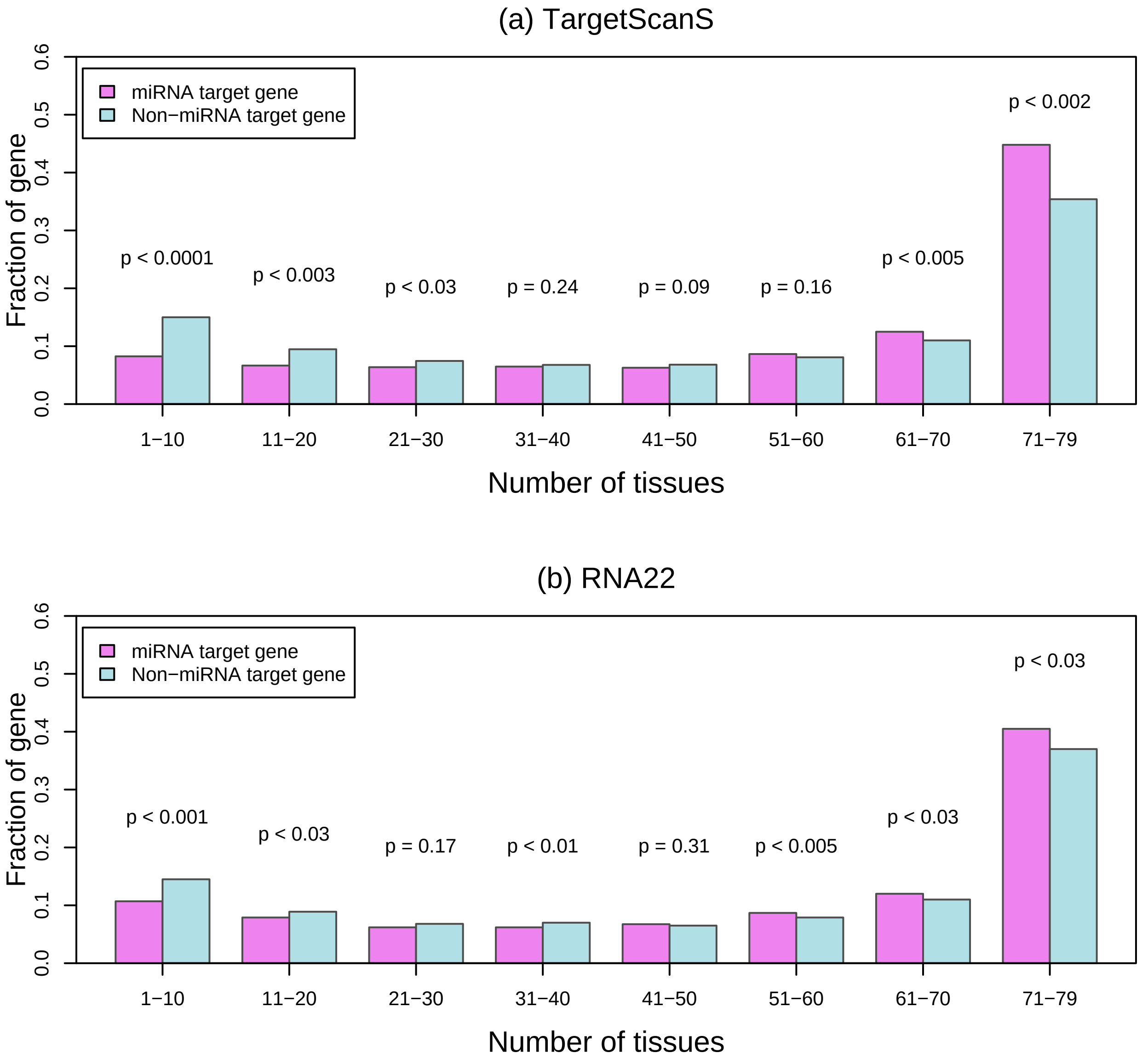

Figure S2. Expression breadth differences between non-miRNA and miRNA target genes predicted from TargetScanS and RNA22. Distribution of the fraction of miRNA and non-miRNA target genes restrictedly expressed in certain number of tissues for miRNA target genes predicted from TargetScanS (a) and RNA22 (b). p-values: statistical differences of median fractions between miRNA and non-miRNA target genes in each bin by Wilcoxon signed rank tests.
