# Supplementary material for: Insight into microRNA regulation by analyzing the characteristics of their targets in humans
Source: BMC Genomics. 2009 Dec 10;10:594. doi: 10.1186/1471-2164-10-594 (PMC2799441; doi:10.1186/1471-2164-10-594)
Supplement: Additional file 3 — Supplemental figure 3. Shows the correlation between mRNA expression and decay rate for miRNA target genes predicted from TargetScanS. [file 1471-2164-10-594-S3.PDF]

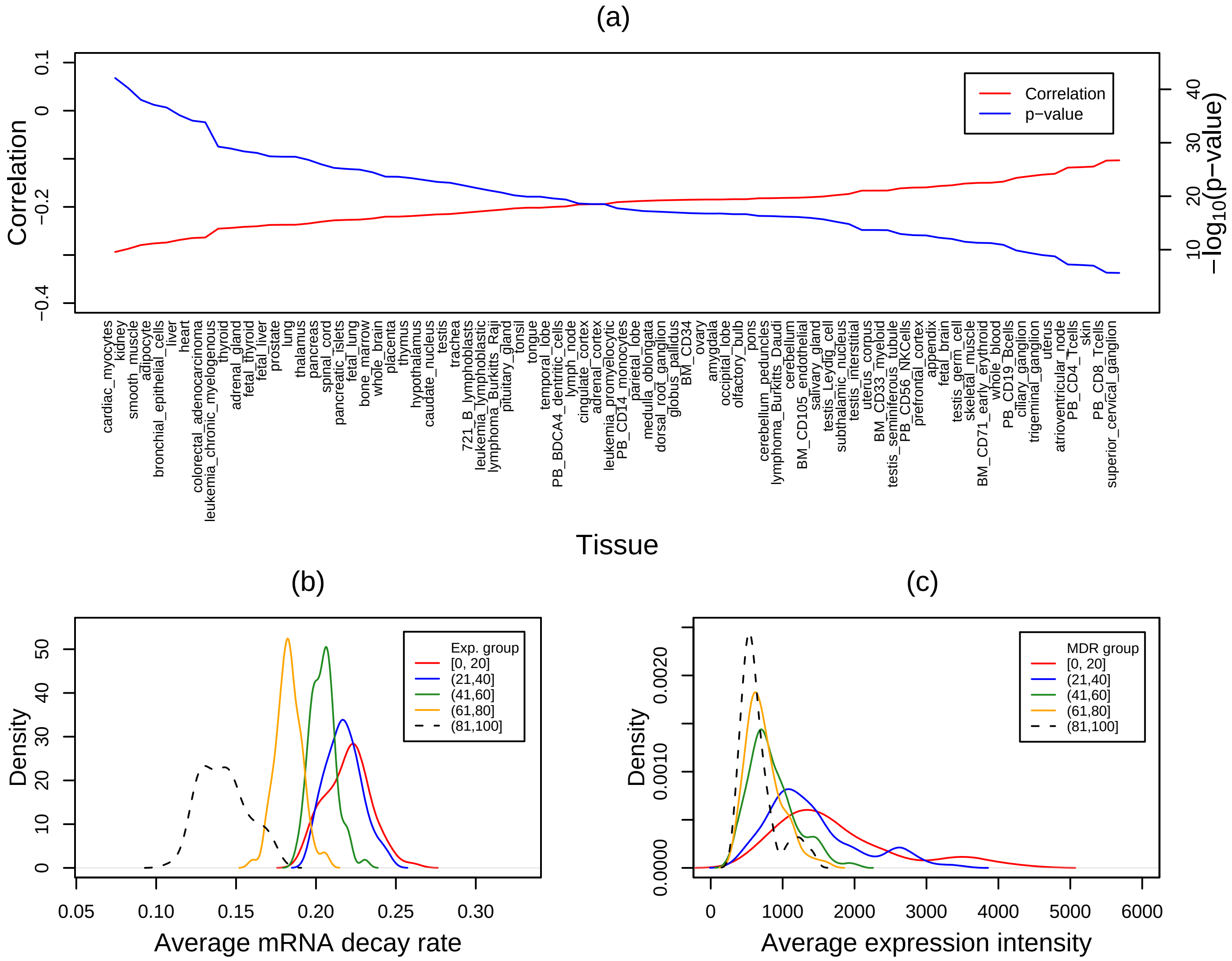

Figure S3. Correlation between mRNA expression and decay rate for miRNA target genes predicted from TargetScanS. (a) Spearman's rank correlation rho between gene expression from each of the 79 human tissues and mRNA decay rates, and corresponding p-values (-log10(p-values)) for the correlation coefficients. (b) Distribution of the average mRNA decay rates, which were obtained from comparing gene expression in each of the 79 human tissues for 5 mRNA expression groups with increasing expression values from the group [1,20] to group (80,100]. (c) Distribution of the average mRNA expression values in the 79 human tissues for 5 mRNA decay rate groups with increasing mRNA decay rate from the group [1,20] to group (80,100]. Exp: mRNA expression; MDR: mRNA decay rate.
