# Supplementary material for: Insight into microRNA regulation by analyzing the characteristics of their targets in humans
Source: BMC Genomics. 2009 Dec 10;10:594. doi: 10.1186/1471-2164-10-594 (PMC2799441; doi:10.1186/1471-2164-10-594)
Supplement: Additional file 5 — Shows the correlation between gene expression and protein stability for miRNA target genes predicted from TargetScanS. [file 1471-2164-10-594-S5.PDF]

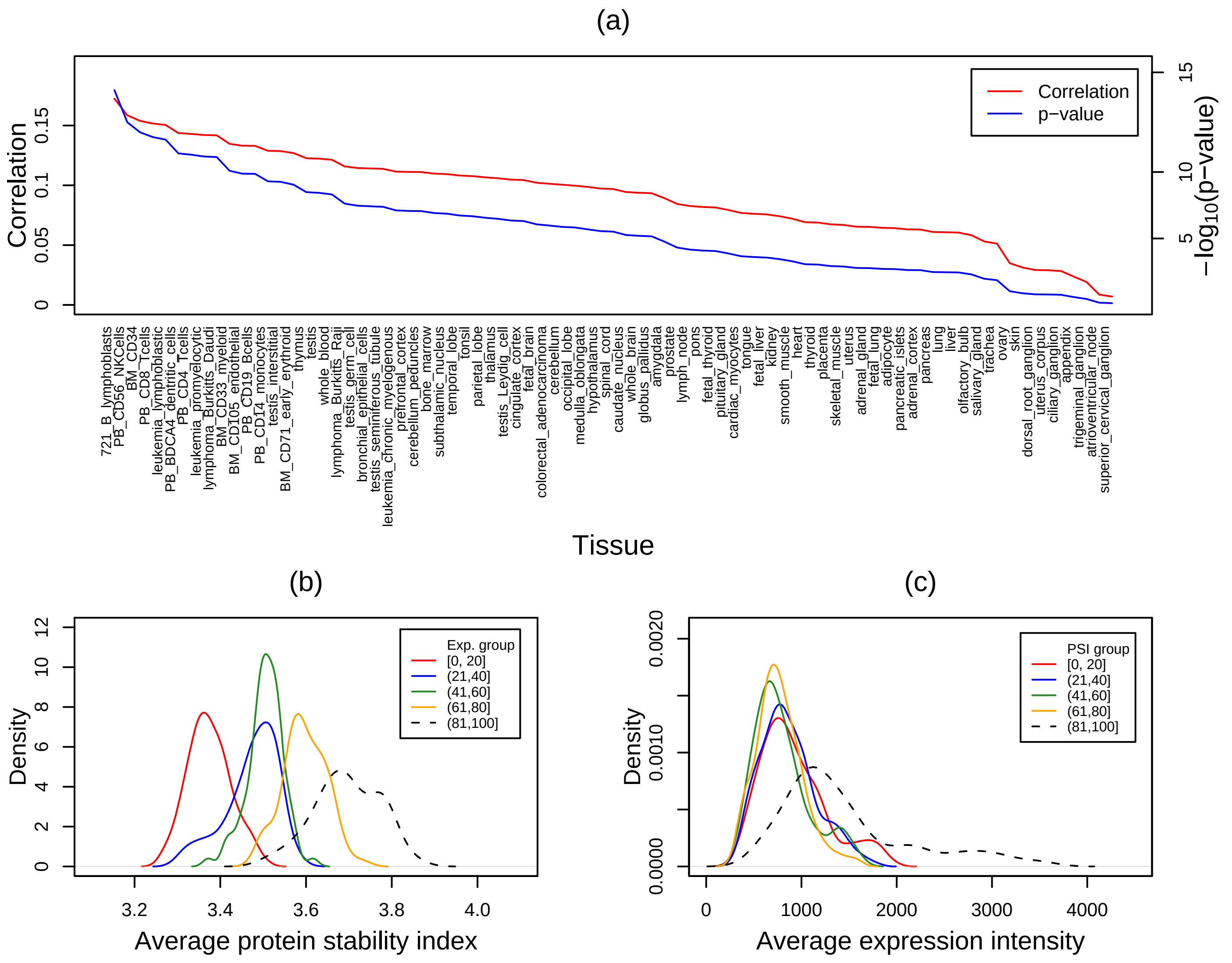

Figure S5. Correlation between mRNA expression and protein stability for miRNA target genes predicted from TargetScanS. (a) Spearman's rank correlation rho between gene expression from each of the 79 human tissues and protein stability, and corresponding p-values (-log10(p-values)) for the correlation coefficients. (b) Distribution of the average protein stability indices, which were obtained from comparing gene expression in each of the 79 human tissues for 5 mRNA expression groups with increasing expression values from the group [1,20] to group (80,100]. (c) Distribution of the average mRNA expression values in the 79 human tissues for 5 protein stability groups with increasing protein stability index from the group [1,20] to group (80,100]. Exp: mRNA expression; PSI: protein stability index.
